# Dual Modification of Starch by Physical Methods Based on Corona Electrical Discharge and Ionizing Radiation: Synergistic Impact on Rheological Behavior

**DOI:** 10.3390/foods11162479

**Published:** 2022-08-17

**Authors:** Mirela Braşoveanu, Monica R. Nemţanu

**Affiliations:** Electron Accelerators Laboratory, National Institute for Laser, Plasma and Radiation Physics, 409 Atomiştilor St., P.O. Box MG-36, Măgurele, 077125 Bucharest, Romania

**Keywords:** apparent viscosity, corn starch, electron beam, irradiation, plasma, synergy ratio

## Abstract

The present paper focuses on evaluating the synergistic effects of dual modification with corona electric discharge (CED) and electron beam irradiation (EBI) on the rheological behavior of starch. Combined treatments were applied successively (CED/EBI and EBI/CED) and compared with single treatments. The outcomes showed that the rheological features of starch were altered by the dual modification in correlation with the irradiation dose mainly as a result of radiation-induced degradation. Decreases in apparent viscosity were described by exponential-like-models according to the order of application of the treatment sequences. The mathematical models allowed the estimation of the irradiation doses for which the viscosity decreased by *e* times for the dual modified starches (3.3 ± 1.3 kGy for CED/EBI and 5.6 ± 0.5 kGy for EBI/CED, respectively) and the fraction (*f*) of 0.47 ± 0.10 corresponding to starch granule considered to be affected by plasma. Both dual treatments yielded a synergistic effect, regardless of the order of application of the treatment sequences, being more effective in decreasing starch apparent viscosity than single EBI. However, synergism evaluation proved that the use of plasma as a pre-treatment to irradiation processing could provide benefits up to 20 kGy. These findings support the practical goals of technologists with valuable information that may facilitate or simplify the experimental design of starch dual modification with plasma and ionizing radiation.

## 1. Introduction

Starch is one of the most popular herbal polymers with a wide range of both food and non-food applications. As nature restricts the sources of starch and thereby its properties, it is therefore necessary to continually advance and upgrade starch modification processing, leading to new properties and applications and thus to improved starch performance [1]. Over time, an impressive number of research papers have sought to reveal solutions to overcome the technological limitations of native starch and thus expand its uses. In this regard, the interest in starch modification by using emerging and non-polluting techniques has grown significantly worldwide [2,3].

Recent trends of starch modification explore various approaches of combining different single modification methods to optimize their functionalities in the dual modification as well as to improve the physicochemical properties of starch [4,5]. Dual modifications can be classified into homogeneous modifications (dual chemical, dual physical, or dual enzymatic modifications) and heterogeneous modifications (combination of different single modification methods, i.e., chemical/physical, chemical/enzymatic) [4,6]. For instance, the combination of different methods, such as phosphorylation/acetylation [7], ultrasonication/high voltage electric field [8], cyclodextrin glycosyltransferase/specific cyclodextrinase [9], heat moisture treatment/octenyl succinylation [10], cross-linking/annealing [11], or debranching/propionate esterification [12], can induce dual modifications of starch from various botanical sources like sago, potato, corn, and rice.

Although chemical methods give more options for starch functionalization and consequently significantly expand starch applications [6], dual physical modification has recently gained increasing interest in terms of “green” or “sustainable” chemistry, because no other chemical is introduced into the starch granules [4]. In recent years, dual physical modification methods based on “green” physical techniques, including ionizing radiation or plasma discharges, have been studied on different starches from rice, corn, mung bean, sago, lentil, talipot, potato, and red adzuki bean. For example, promising and extensive works on the combination of physical methods such as microwaves/cold plasma [13], gamma irradiation/pyrolysis [14], corona electrical discharge plasma/electron beam irradiation [15], ultrasound/dielectric barrier discharge plasma [16], gamma irradiation/annealing [17], sonication/gamma irradiation [18,19], twin-screw extrusion/cold plasma [20], and dry heat/ dielectric barrier discharge plasma [21] have been conducted by research groups around the world. The reported results revealed that the changes in starch functionalities appear to be caused by degradation or cross-linking effects, which may depend on the processing parameters, application sequences of the studied treatments, and starch characteristics.

In line with this trend, we addressed the topic of the dual modification of starch by applying the combined action of corona electric discharge with ionizing radiation, namely the electron beam, in order to lower the minimum dose of irradiation necessary for technological applications in starch processing. In this regard, in our previous work [15], we revealed that the combined action of the corona electric discharge (CED) and electron beam irradiation (EBI) can cause more noticeable alterations of starch features such as acidity, paste clarity, gel consistency, and viscosity for the same irradiation dose than the single EBI, determined by the sequence of CED application, as a result of the degradation phenomenon. Moreover, it was demonstrated that the usage of CED as a pre-treatment to EBI of the starch had the highest efficiency in modifying the viscosity among the combined sequences of the single methods. Therefore, the current paper is planned to be a continuation of the previous study [15] which was initiated on this topic of the dual physical modification of starch. In the present work, we focused on the impact of dual modification with CED and EBI on the rheological behavior of starch, taking into account that the rheological aspects of starch are an important component of any experimental starch design. In addition, the viscosity is one of the most important rheological properties in most production stages of a product, and even more so, it has a direct impact on the quality of the final product. It should be noted here that several of the studies on dual changes in starch [9,21,22,23,24,25] mention that a combination of processing methods has a synergistic effect on starch. Synergism means more than the sum effect of two or more methods when applied individually. Although, in the literature, there are such qualitative comments and highlights of the dual modification of starch, there is still no study to date that explores this issue and clearly, quantitatively, and responsibly proves such a synergistic effect, regardless of the investigated combined methods. This raises the question of whether the intuitive, qualitative assumption of synergistic impact is sufficient or whether more is needed when the studies are carried out with the intention of their practical application.

In our previous study [15], we detected a possible synergistic effect of the combined action of CED and EBI in terms of the rheological element of starch, which prompted us to further investigate and quantify this potential synergism. Such an approach can support the practical intentions of technologists through valuable information, which can facilitate or simplify the experimental design of the dual modification of starch with plasma and ionizing radiation.

Therefore, this paper aimed to analyze and highlight in detail the rheological effects of dual modification of starch by combining methods involving CED and EBI. An assessment of the synergistic effect from a quantitative perspective was also considered.

## 2. Materials and Methods

### 2.1. Materials

Native corn starch (S4126; 11% moisture; ~73% amylopectin and 27% amylose) employed in the experiments was acquired from Sigma-Aldrich Company (St. Louis, MO, USA).

### 2.2. Exposure to Corona Electrical Discharge (CED)

Experiments were performed in a repetitive pulsed electrical discharge set-up that has been disclosed elsewhere [26,27]. The operating parameters were as follows: current intensity of 45 A; electrical pulses of 25 kV amplitude and 50 Hz pulse repetition frequency with a pulse duration of 100 ns. Starch in its granular form was exposed to CED in air, at ambient pressure, for 5 min. Untreated (native) starch was regarded as the control sample. After treating the starch, the samples were kept in the dark and at room temperature (23 ± 1 °C).

### 2.3. Electron Beam Irradiation (EBI)

Packed starch samples (4.5 cm × 4.5 cm) were irradiated with an electron beam generated by the linear accelerator ALIN-10 (NILPRP, Bucharest-Măgurele, Romania). This electron accelerator generates an electron beam with an energy of 6.23 MeV and peak current of 75 mA for a fixed pulse duration of 3.5 μs and repetition frequency of 100 Hz [28]. The ALIN-10 accelerator is a laboratory installation used in different radiation researches [29,30,31,32]. For the experiments in this work, the irradiations were performed in static mode at room temperature (23 ± 1 °C) and ambient pressure in air. The irradiation doses were in the range of 10–50 kGy with a mean dose rate of 2 kGy/min. Non-irradiated (native) starch was regarded as the control sample. After irradiations, the samples were kept in the dark and at room temperature (23 ± 1 °C).

### 2.4. Corona Electrical Discharge in Conjunction with Electron Beam Irradiation (CED/EBI)

Starch samples were first treated with corona electrical discharge in air for 5 min as described above in Section 2.2. Further, the samples were exposed to irradiation in the same conditions as depicted earlier in Section 2.3. After treatments, the samples were kept in the dark and at room temperature (23 ± 1 °C).

### 2.5. Electron Beam Irradiation Combined with Corona Electrical Discharge (EBI/CED)

Starch samples were subjected to EBI, similarly to the description in Section 2.3, followed by the exposure to CED in air for 5 min, as explained in Section 2.2. After treatments, the samples were kept in the dark and at room temperature (23 ± 1 °C).

### 2.6. Rheological Measurements

Rheological evaluation was carried out on starch samples by using a VT^®^ 550 rotational viscosimeter (ThermoHaake, Germany) with a NV coaxial cylinder, as described in our previous work [15]. The shear stress, *τ*, and the apparent viscosity, *η_a_*, were measured on 5% (*w*/*v*) starch samples at different shear rate values up to 541 s^−^^1^ at room temperature (25 ± 1 °C). The Ostwald de Waele rheological model (Equation (1)), which is mathematically known as the power law equation, was selected to fit the viscosity behavior in this work. The Ostwald de Waele model is the most frequent and reliable two-parameter model used in engineering applications to describe the relationship between the shear stress, *τ*, and the shear rate, $\dot{\gamma }$, of non-Newtonian fluids.

(1) $$\tau =k\cdot {\dot{\gamma }}^{n}\;\mathrm{or}\;\eta_{a}=k\cdot {\dot{\gamma }}^{n-1}$$

where *τ* is the shear stress (Pa), *k* is the fluid consistency coefficient (Pa∙s^n^), *n* is the flow behavior index (dimensionless), $\dot{\gamma }$ is the shear rate (s^−1^), and *η_a_* is the apparent viscosity (Pa∙s).

### 2.7. Statistical Approach

The results reported are presented as mean values ± standard deviation of triplicate determinations. For data processing, OriginPro 8.1 (OriginLab Corporation, Northampton, MA, USA), Microsoft^®^ Excel 2010 (Microsoft Corporation, Redmond, WA, USA), and InfoStat versión 2020 [33] were used. The data analysis was performed by using an analysis of variance with the Fisher LSD (least significant differences) post-hoc test to discern the statistical difference. A probability value *p* ≤ 0.05 was considered statistically significant.

## 3. Results and Discussion

### 3.1. Flow Behavior

**Figure 1:**
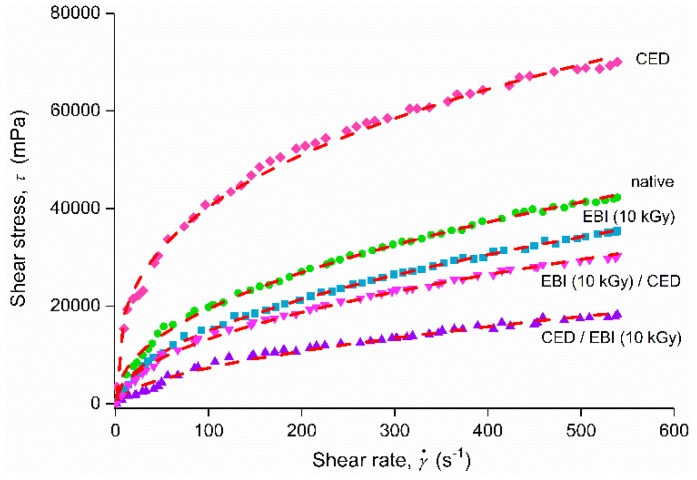
Flow curves of native and treated samples—fitted with the Ostwald de Waele model.

The native starch had a non-Newtonian behavior (Figure 1), showing that the ratio of the shear stress and shear rate was not constant, and the shear stress dependence of shear rate indicated the shear-thinning (pseudoplastic) character. According to Park et al. [34], this behavior can be the result of the breaking of the entangled macromolecule network during shearing.

The exposure of native starch to a single CED treatment affected this behavior, although the modified starch kept the non-Newtonian character. On the contrary, the single EBI of native starch also induced changes in this behavior, making it more Newtonian-like, with the increasing irradiation dose. Furthermore, the application of combined treatments to starch further altered the non-Newtonian behavior as the irradiation dose increased in comparison to single EBI. As an example, Figure 1 displays the rheograms for single and combined treatments applied to starch, at the same irradiation dose of 10 kGy.

**Figure 2:**
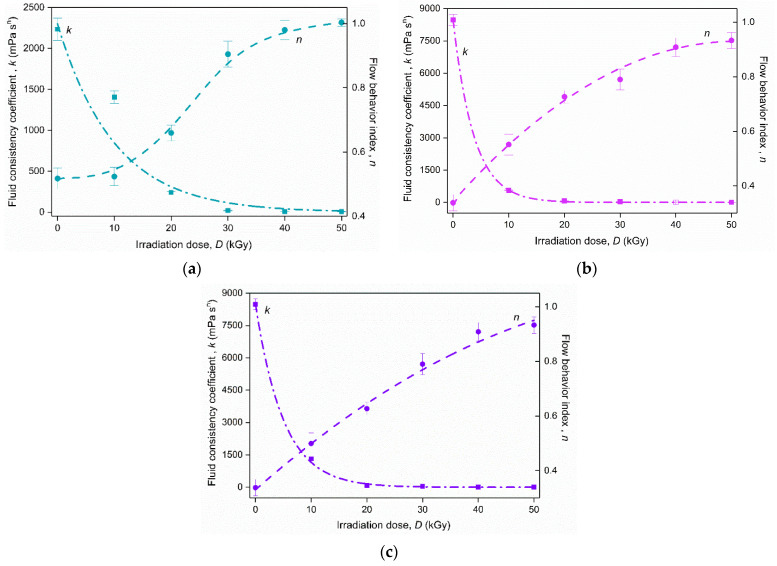
Rheological parameters of the Ostwald de Waele model for samples exposed to (**a**) EBI, (**b**) CED/EBI, and (**c**) EBI/CED.

In the present investigation, the apparent viscosity of native and modified starch samples was fitted with the Ostwald de Waele model with a high coefficient of determination (*R*^2^ ≥ 0.960). The resulting rheological parameters, *n* and *k*, respectively, are presented in Figure 2. The flow behavior index values confirmed the pseudoplastic behavior as they deviated from 1 (*n* < 1). As is well known, the lower the value of the flow index, the greater is the degree of shear-thinning [35]. Therefore, it can be noticed that the samples treated with both single EBI and combined treatments had less of a shear-thinning character, with a trend towards Newtonian one (*n* = 1) as the irradiation dose increased. Such behavior indicates that samples could undergo a degradation phenomenon after the treatments. This finding was confirmed by the evolution observed for the consistency coefficient, *k*, which also had a decreasing trend with increasing irradiation dose, indicating the tendency of the sample to flow more easily as the irradiation dose increased.

### 3.2. Apparent Viscosity

The apparent viscosity value (25 °C, $\dot{\gamma }$ = 200 s^−1^) of the native starch sample increased (*p* ≤ 0.05) after CED treatment. This result may be attributed to a cross-linking phenomenon that occurred in the granular starch exposed to CED under the experimental conditions selected herein. The finding is congruent with previous reports [15,36]. Kartha and Srivastava [37] proved that the peak and cold paste viscosities increased by introducing even low degrees of cross-linking in starch. Similarly, another study [38] revealed that the cross-linked rice and maize starches showed increased peak and final viscosities, in contrast to the potato cross-linked starch, which had a reduction in the peak and final viscosities. In the same way, Kou and Gao [39] showed that cross-linked corn and potato starches had higher peak and final viscosities in comparison with their native counterparts, in contrast to the pea cross-linked starch that had viscosity values lower than the native form. Thus, under the same cross-linking condition, some starches show an increase of their viscosities due to the low degree of cross-linking, whereas other starches can have low viscosities as a result of a higher extent of cross-linking. In fact, Shah et al. [40] explained in detail the effect of cross-linking on starch viscosity behavior, showing clearly that the peak viscosity of cross-linked starch was higher than that of native starch, whereas starch with a higher level of cross-linking showed a lower peak viscosity unlike starch with a low level of cross-linking. Very recently, Zhang et al. [41] also found that cross-linking reactions induced an increase in the viscosities of tapioca starch exposed to low-pressure plasma under different feeding gases.

Although the most important chemical mechanism of polymer exposure to corona discharge is oxidation, the cross-linking of molecules on the surface can also occur and further limit their mobility, leading to an increase in molecular weight [42,43] with an effect on the viscosity value. The application of CED to polymeric materials leads to the formation of free radicals that further react with oxygen or residual water, giving peroxy radicals. The peroxy radicals are able to induce opposite reactions, chain scission accompanied by molecular weight reduction, or cross-linking accompanied by a molecular weight increase, depending on the power level [42,43]. Moreover, Wongsagonsup et al. [44] explicitly proved that factors such as sample preparation and input power play crucial roles in determining which competitive reaction is dominant for starch modification by plasma. In addition, Deeyai et al. [45] reported that the degree of cross-linking of starch exposed to plasma also depended on relative humidity, so that it increased with decreasing the moisture content of starch. Even more, the gas composition can also influence the cross-linking degree of starch exposed to plasma processing [41].

**Figure 3:**
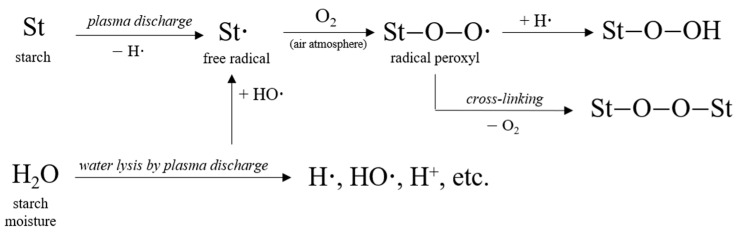
Cross-linking mechanism for plasma-processed starch.

**Figure 4:**
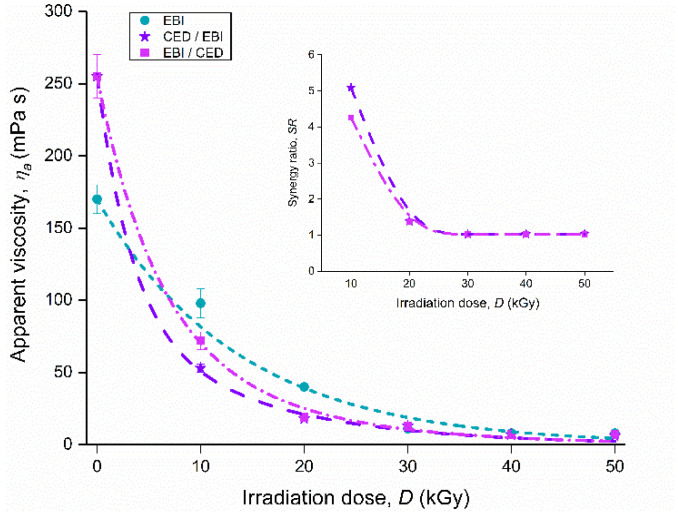
Evolution of the apparent viscosity (25 °C, $\dot{\gamma }$= 200 s^−1^) for modified starches depending on the irradiation dose. Inner window: Synergy ratio vs. irradiation dose for combined treatments (EBI/CED and CED/EBI).

**Figure 5:**
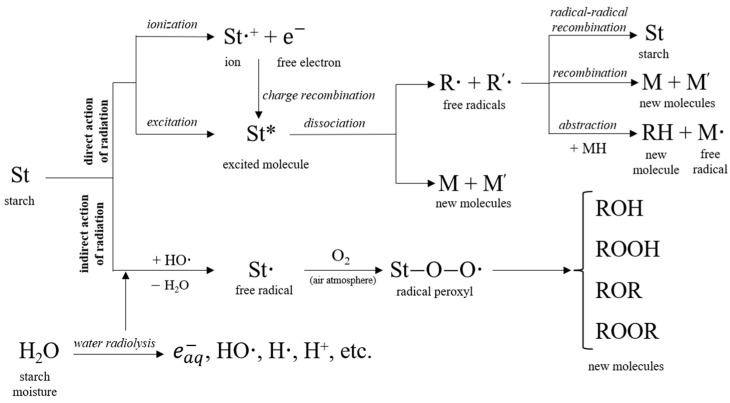
Oxidative degradation mechanism for radiation-processed starch.

Taking into account the basic concepts and current knowledge in polymer and plasma science, we suggest a possible mechanism of cross-linking for starch exposed to plasma processing in the air atmosphere as below (Figure 3):

Conversely, the apparent viscosity (25 °C, $\dot{\gamma }$ = 200 s^−1^) of the native starch sample exposed to EBI had a decreasing evolution (*p* ≤ 0.05) with the increase of the irradiation dose (Figure 4). This behavior may be assigned to the degradation of the starch molecular structure to lower molecular weight structures. The observation is in agreement with other previous reports on EB-irradiated starch and its components [15,46,47,48]. The changes that occur in starch by exposure in the presence of atmospheric oxygen to high doses of the electron beam are the result of the direct action of radiation on the starch and indirect action due to the radiolysis of starch moisture content. The direct action by ionization and excitation of atoms and molecules leads to free radicals, which further participate in other recombination processes. Indirectly, the hydrogen and hydroxyl radicals formed by radiolysis of water molecules in native starch attack macromolecules, producing their macroradicals, which are implicated in free radical reactions, followed by chain scission and even glucose ring opening. For amylose, one of the main components of starch, it has been proven that it can undergo a degradation phenomenon by random main-chain scission when it is exposed in the solid state to EBI, in the presence of oxygen, in the range of 10–50 kGy [48]. The disruption of the amylose structure thus led to the appearance of smaller fragments as a result of irradiation. Therefore, lower molecular weight structures may be formed, leading to a decrease in starch viscosity. Based on literature reports about the reactions of polymers in general and starchy materials in particular under ionizing radiation [49,50,51,52,53,54], we propose herein a possible mechanism of oxidative degradation of starch under the radiation action (Figure 5):

In order to bring better clarity in the description of the effects of the investigated dual treatments, regardless of the order of the application sequence of the single methods, we specify here that the samples treated with the irradiation dose *D* = 0 are practically the samples exposed only to plasma as a pre-treatment or as a post-treatment of EBI. In other words, this means that the viscosity of the samples treated with the irradiation dose *D* = 0 is the same as the viscosity of the starch treated only with CED alone for both CED/EBI and EBI/CED.

The CED/EBI treatment of the samples caused a greater decrease (*p* ≤ 0.05) in apparent viscosity for doses up to 40 kGy in comparison with single EBI (Figure 4). This result indicates that the effect induced in the starch molecule by EBI was enhanced by the CED pre-treatment. This may be due to the phenomenon of cross-linking (formation of tridimensional network) caused by corona discharge at the surface of the macromolecule, which then blocked the presence of oxygen found in the working atmosphere when irradiation occurred. According to Kamal et al. [55], oxygen may exert a kind of mild protection of the molecule, reducing the degradation yield when starch was irradiated in oxygen-saturated atmosphere. Thus, we can practically consider that only high energy electrons and radiolysis radicals caused degradation of the granule content concurrently with the attack of the cross-linked network from the surface, leading to a double damage with a stronger total effect than in the case of single irradiation. Results previously published by other research groups show, almost similarly, the reduction of starch viscosities due to dual treatments of cross-linking followed by oxidation [38], hydrolysis [56], microwave irradiation [57] or heat moisture treatment [58]. In this respect, it was demonstrated that when starch with a certain degree of cross-linking is subsequently exposed to a degradation treatment, lower viscosities occurred in the pasting profile compared to the native starch, except for peak and breakdown viscosities in the case of oxidation or setback and final viscosities in the case of heat moisture treatment.

On the other hand, the samples exposed to the EBI/CED treatment also showed a reduction (*p* ≤ 0.05) in the apparent viscosity values with the increase of the irradiation dose (Figure 4). In this case, it can be noticed that these values were generally lower (*p* ≤ 0.05) than those induced by EBI up to 40 kGy but higher than those caused by CED/EBI for irradiation doses up to 20 kGy. Under these circumstances, the initial application of the irradiation sequence determined the oxidative degradation of the starch architectural structure, resulting in a new material (single modified starch). The subsequent exposure of this irradiated starch to a plasma treatment sequence enhanced the degradation without internal penetration but only at the surface. It is notorious that polysaccharides are prone to the degradation phenomenon under radiation. In other words, the high energy electrons (of the MeV order) caused a massive degradation of the starch in the studied dose range, while the ions and electrons of lower energy (of the eV order) of the plasma applied afterwards produced only a superficial degradation. The overall effect of this viscosity-reducing treatment was found to be stronger than single EBI, but less effective than the CED/EBI combination.

### 3.3. Modeling of Apparent Viscosity Trend

Ionizing radiation (electron beam or gamma radiation) can cause an exponential decrease in the native starch viscosity as the irradiation dose increases [15,30,47]. Thus, in our case, the exponential law of the apparent viscosity decreasing by EBI can be expressed as follows:

(2) $$\eta_{a}^{EBI}=\eta_{a0}e^{-D/D_{EBI}}$$

where $\eta_{a}^{EBI}$ is the apparent viscosity of the starch (mPa·s) irradiated with irradiation dose *D* (kGy), $\eta_{a0}$ is the apparent viscosity of the native starch ($\eta_{a0}$ = 170 ± 10 mPa∙s), and $D_{EBI}$ is the characteristic irradiation dose (kGy).

As defined by Nemtanu and Brasoveanu [47], the material constant $D_{EBI}$, resulting from the exponential decreasing law of the apparent viscosity against the irradiation dose, is the characteristic irradiation dose of starch for which the viscosity decreases by *e* times. In other words, this constant is a material characteristic that quantitatively describes the intrinsic ability of a starch to degrade in the field of ionizing radiation (electron beam or gamma radiation). Such a parameter is useful both to evaluate the sensitivity to degradation of different starches subjected to irradiation and to estimate the irradiation dose required to achieve the viscosity value of interest in an experimental setup or technological process optimization. Considering these aspects in the present study, the estimated mean value of $D_{EBI}$ was 11.9 ± 1.6 kGy (*R*^2^ = 0.9840) for starch modified by EBI. This result is in accordance with previously reported studies [30,47], where the characteristic irradiation dose for corn starch exposed to ionizing radiation was found to be in the range of 9–15 kGy.

**Figure 6:**
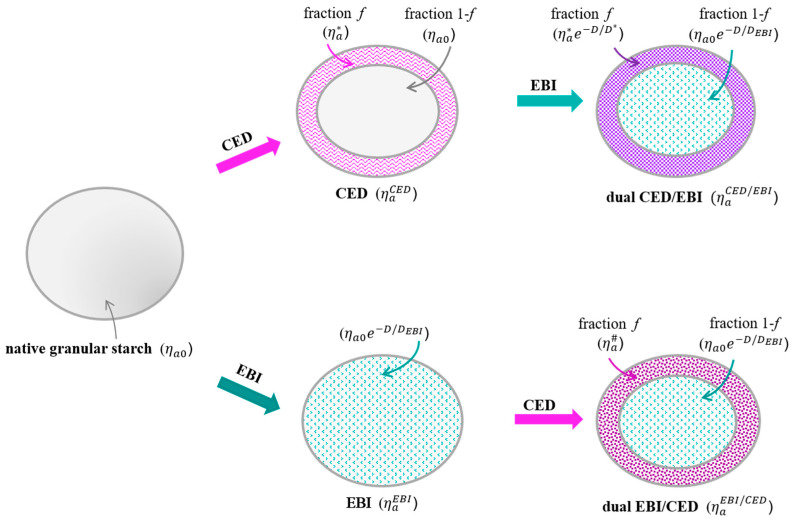
Schematic representation of the investigated treatments applied to the starch.

The combined treatments also affected the decrease of the apparent viscosity in an exponential manner with the irradiation dose but imposed a modeling of its evolution according to a more complex law. In this regard, to describe the evolution of the viscosity of the starch modified by combined treatments, we considered that only a fraction (*f*) of granular starch can actually be affected by exposure to CED (Figure 6). This hypothesis took into account that the plasma processing of biodegradable polymers generally leads to various surface modifications [59]. Thus, after exposure to CED, the fraction (*f*) is characterized by a viscosity, $\eta_{a}^{*}$, while the unaffected fraction (1 − *f*) keeps the same viscosity as before the exposure of starch to CED.

#### 3.3.1. CED/EBI

When the native starch having the viscosity $\eta_{a0}$ was exposed to CED as a pre-treatment, the viscosity of the pre-treated starch, $\eta_{a}^{CED}$, followed the Equation (3):

(3) $$\eta_{a}^{CED}=\left(1-f\right)\eta_{a0}+f\eta_{a}^{*}$$

where $\eta_{a}^{CED}$ is the apparent viscosity measured after exposure to CED ($\eta_{a}^{CED}$ = 255 ± 15 mPa·s), *f* is the fraction of the starch granule affected by plasma, $\eta_{a0}$ is the apparent viscosity of the native starch ($\eta_{a0}$ = 170 ± 10 mPa∙s), and $\eta_{a}^{*}$ is the apparent viscosity of the starch fraction (*f*) affected by CED (mPa·s).

After exposure to plasma, both the affected fraction (*f*) and the unaffected fraction (1 − *f*) each had a specific response to EBI and contributed differently to the apparent viscosity, $\eta_{a}^{EBI/CED}$, of the starch treated by the CED/EBI method. Therefore, the unaffected fraction (1 − *f*) responded to the EBI with the characteristic irradiation dose $D_{EBI}$ resulting from Equation (2). On the other hand, the fraction (*f*) responded to EBI with the characteristic irradiation dose, $D^{*}$, so that the apparent viscosity of the starch pretreated in CED and then exposed to EBI, $\eta_{a}^{CED/EBI}$, followed an evolution with irradiation dose as below:

(4) $$\eta_{a}^{CED/EBI}=\left(1-f\right)\eta_{a0}e^{-D/D_{EBI}}+f\eta_{a}^{*}e^{-D/D^{*}}$$

where $\eta_{a}^{CED/EBI}$ is the apparent viscosity measured after exposure to CED ($\eta_{a}^{CED/EBI}$ = 255 ± 15 mPa·s), *f* is the fraction of the starch granule affected by plasma, $\eta_{a0}$ is the apparent viscosity of the native starch ($\eta_{a0}$ = 170 ± 10 mPa∙s), $\eta_{a}^{*}$ is the apparent viscosity of the starch fraction (*f*) affected by CED (mPa·s), *D* is the irradiation dose (kGy), $D_{EBI}$ is the characteristic irradiation dose (kGy), and $D^{*}$ is the characteristic irradiation dose of the starch fraction (*f*) affected by CED (kGy).

Thus, if we consider $\eta_{a}^{*}$ from Equation (3), then Equation (4) may be written as such:

(5) $$\eta_{a}^{CED/EBI}=\left(1-f\right)\eta_{a0}e^{-D/D_{EBI}}+\left[\eta_{a}^{CED}-\left(1-f\right)\eta_{a0}\right]e^{-D/D^{*}}$$

By fitting the experimental data, it was found that the characteristic irradiation dose of the fraction (*f*) affected by CED had the value $D^{*}$ = 3.3 ± 1.3 kGy (*R*^2^ = 0.9304). At the same time, the fraction (*f*) had a value of 0.47 ± 0.10, indicating that only this fraction of the total starch amount was affected by plasma exposure. The fraction (*f*), affected by CED pre-treatment, thus became more sensitive to the EBI and had a lower characteristic irradiation dose compared to the mean characteristic irradiation dose of the native starch directly exposed to EBI, with $D_{EBI}$ = 11.9 ± 1.6 kGy.

#### 3.3.2. EBI/CED

In the case of the second type of combined treatment, the native starch was first exposed to EBI, and the viscosity behavior evolved according to Equation (2). Then, by exposure to CED, only a fraction (*f*) was affected, and the fraction (1 − *f*) continued to be characterized by the viscosity given by EBI. Equation (6) describes this evolution:

(6) $$\eta_{a}^{EBI/CED}=\left(1-f\right)\eta_{a}^{EBI}+f\eta_{a}^{\#}$$

where $\eta_{a}^{EBI/CED}$ is the apparent viscosity of the starch treated with EBI/CED (mPa·s), $\eta_{a}^{EBI}$ is the apparent viscosity of the starch (mPa·s) irradiated with irradiation dose *D* (kGy), *f* is the fraction of the irradiated starch granule affected by plasma, and $\eta_{a}^{\#}$ is the apparent viscosity of the irradiated starch within fraction (*f*) affected by CED (mPa·s).

It is noteworthy here that the model contains the product of fraction (*f*) and $\eta_{a}^{\#}$ and cannot disclose their contributions to the viscosity $\eta_{a}^{EBI/CED}$, determined by the affected starch fraction and its corresponding viscosity value. The EBI starch basically becomes a new material that responds to plasma processing in relation to the irradiation dose *D* and a material constant, which is a characteristic of the processes involved. Furthermore, we assumed that fraction (*f*) was the same for all investigated irradiation doses. Thus, the apparent viscosity of the starch treated with combined EBI/CED, $\eta_{a}^{EBI/CED}$, could then be described by an equation as below:

(7) $$\eta_{a}^{EBI/CED}=\left(1-f\right)\eta_{a0}e^{-D/D_{EBI}}+f\eta_{a}^{*}e^{-D/D^{\#}}$$

where $\eta_{a}^{EBI/CED}$ is the apparent viscosity of the starch treated with EBI/CED (mPa·s), *f* is the fraction of the irradiated starch granule affected by plasma, $\eta_{a0}$ is the apparent viscosity of the native starch ($\eta_{a0}\;$= 170 ± 10 mPa∙s), $\eta_{a}^{*}$ is the apparent viscosity of the starch fraction (*f*) affected by CED (mPa·s), *D* is the irradiation dose (kGy), $D_{EBI}$ is the characteristic irradiation dose of the native starch (kGy), and $D^{\#}$ is the characteristic irradiation dose of the irradiated starch within fraction (*f*) affected by CED (kGy).

This Equation also satisfies the condition in Equation (3), when *D* = 0.

For this treatment, by fitting the experimental data, it was obtained that the characteristic irradiation dose of the fraction (*f*) affected by CED within the irradiated starch had the value $D^{\#}$= 5.6 ± 0.5 kGy (*R*^2^ = 0.9985). Thus, it was noticed that $D^{\#}$ was significantly higher compared to the characteristic irradiation dose $D^{*}$ = 3.3 ± 1.3 kGy of the fraction (*f*) obtained for the previous combined treatment, which involves starch pretreatment in CED. At the same time, in this case, the fraction (*f*) affected by plasma treatment had a lower characteristic irradiation dose (*p* ≤ 0.05) than the mean characteristic irradiation dose of the native starch directly exposed to EBI, $D_{EBI}$ = 11.9 ± 1.6 kGy.

### 3.4. Synergistic Effect

It is well known that a synergistic effect is the result of two or more processes interacting together to produce an effect greater than the cumulative effect that those processes produce when used individually.

To prove the synergy, we considered a synergy ratio, *SR*, which is the ratio between the observed and predicted effects [60] of the joint action of the studied physical treatments on the apparent viscosity:

*SR* = *E_obs_*/*E_pr_* (8)

where *E_obs_* and *E_pr_* are percentages of the observed effect and predicted effect, respectively.

The predicted effect of corona discharge pre-treatment in conjunction with accelerated electrons (CED/EBI) on native starch was estimated using Abbott’s formula [61]:

*E_pr_* = *E_CED_* + *E_EBI_* − (*E_CED_* × *E_EBI_*/100) (9)

in which the level of effect of each single treatment, *E_CED_* and *E_EBI_*_,_ respectively, was calculated using the following formulas:

(10) $$E_{CED}=\left[\left(\eta_{a0}\;-\eta_{a}^{CED}\right)\;/\eta_{a0}\right]\;\times \;100$$

(11) $$E_{EBI}=\left[\left(\eta_{a0}\;-\eta_{a}^{EBI}\right)\;/\eta_{a0}\right]\times \;100$$

where *E_CED_* and *E_EBI_* are the percentage of effects given by the single treatment, CED and EBI, respectively; $\eta_{a0}$ is the measured apparent viscosity of the native starch (mPa·s); and $\eta_{a}^{CED}$ and $\eta_{a}^{EBI}\;$ are the apparent viscosities of the samples treated with single CED and single EBI at irradiation dose *D*, respectively.

The observed effect of combined treatment (CED/EBI) was calculated similarly to the single treatment effect by using Equation (12), in which $\eta_{a}^{CED/EBI}$ is the measured apparent viscosity of the starch sample subjected to CED/EBI treatment at irradiation dose *D*.

(12) $$E_{obs}=[(\eta_{a0}\;-\eta_{a}^{CED/EBI})\;/\eta_{a0}]\;\times \;100$$

The limits for *SR* were considered as follows:

*SR* > 1—synergistic effect,
*SR* = 1—additive effect,
*SR* < 1—antagonistic effect.

Calculating the synergistic ratio *SR* for each EB irradiation dose in conjunction with CED pre-treatment (CED/EBI), it was observed that all values were greater than 1 (the inset of Figure 4). However, the values decreased (*p* ≤ 0.05) as the EB irradiation dose increased to 30 kGy. The synergistic effect diminished after irradiation with 20 kGy and became practically insignificant (*p* > 0.05) at doses above 30 kGy. According to Kosman and Cohen [60], the value of *SR* reflects the relative intensity of the joint action: the higher the *SR* than 1, the stronger the synergism. Thus, it was clearly demonstrated that CED pre-treatment of starch subsequently exposed to EBI yielded a synergistic effect. In other words, this combined treatment (CED/EBI) was more effective in decreasing the apparent viscosity of corn starch than single EB irradiation.

Similarly, the synergy ratio, *SR*, was calculated for the EBI/CED combined treatment. In this case, the *SR* followed the same pattern with super-unit values as in the previous combination treatment (the inset of Figure 4). Comparing the two combined treatments, there was a notable difference only for the 10 kGy dose, for which *SR* had a higher value (*p* ≤ 0.05) for CED/EBI treatment, indicating that the effect of combining these two techniques (corona electrical discharge and electron beam irradiation) on the starch apparent viscosity was stronger when CED was used as a pre-treatment. This finding is in line with the previous discussions [15], which already showed that CED/EBI treatment would require a lower irradiation dose than single EBI and even EBI/CED treatment to achieve the same viscosity effect.

Therefore, the CED/EBI treatment caused the highest synergy in this study, which can be explained by double damages in the fraction f as a result of the degradation of granule content that occurred through the action of high-energy electrons and radiolysis radicals due to EBI simultaneously with attacking the cross-linking network from the surface formed by CED pre-treatment. Instead, the EBI/CED treatment generated a severe oxidative degradation of the starch structure through the EBI sequence, leading to a modified starch that was no longer able to participate in the cross-linking reaction when the CED sequence was applied. The reactive species of CED practically determined only a slight degradation at the pre-irradiated starch surface. Thus, the CED/EBI treatment of starch modification was based only on the phenomenon of starch degradation, both by EBI and by CED.

## 4. Conclusions

The rheological behavior of starch, especially its key parameter (apparent viscosity), as well as the synergism of the combined CED and EBI methods applied to modify native starch were investigated and evaluated in comparison with the corresponding single methods, in the irradiation dose range of 10–50 kGy. The main outcomes of this study can be outlined as follows:

1. The dual modification affected the starch fluid behavior in a dose-dependent manner. All dual modified samples showed more easy flow with the trend towards the Newtonian flow as the irradiation dose increased, regardless of the treatment application sequence, indicating a degradation effect induced by dual methods investigated.
2. The evolution of the apparent viscosity with the irradiation dose for each investigated treatment could be described by mathematical models that illustrate the exponential-like decrease of the viscosities with increasing irradiation dose.
3. The mathematical estimation of the characteristic irradiation doses for which the viscosity decreases by *e* times for starches modified in dual mode (CED/EBI and EBI/CED) as well as the fraction (*f*) of the starch granule considered to be affected by plasma clearly proved that CED/EBI was a more effective dual modification treatment than EBI/CED.
4. The quantification of the synergistic effect based on the synergistic ratio, *SR*, demonstrated that the plasma pre-treatment of the native starch exposed further to radiation processing (CED/EBI) was more effective in decreasing starch apparent viscosity than the combined treatment involving post-irradiation plasma processing (EBI/CED) up to 20 kGy.
5. The synergistic viscosity-lowering effects occurred due to the joint result of plasma-induced cross-linking and radiation-induced degradation in the case of CED/EBI treatment, but only the degradation phenomenon in the case of EBI/CED treatment.

Thus, the dual modification with corona discharge and ionizing radiation is an efficient tool for the production of starch with modified rheological properties without any introduction of additional chemicals at irradiation doses lower than 20 kGy.

Further studies on this topic should approach structural investigation to better understand and clarify the mechanisms of joint action of the CED and EBI. Furthermore, similar research on starches from different botanical sources or with different features (i.e., moisture content, amylose/amylopectin ratio, crystallinity level), as well as using different experimental conditions (i.e., gas atmosphere, dose rate), should be developed in the future to refine and validate the mathematical models proposed herein for combined methods.

## Data Availability

Data sharing is not applicable for this article.
